# The Workmen's Compensation Act, 1906

**Published:** 1907-02-02

**Authors:** 


### THE WORKMEN'S COMPENSATION ACT, 1906.

BY A MEMBER OF THE BAR.

This Act, which comes into operation on July 1 of the present year, repeals the Workmen's Compensation Acts of 1897 and 1900, and, while re-enacting certain parts of them, very greatly extends the principle which underlies them both. The new Act consists of seventeen sections, many of them very long and complicated, and three schedules dealing with the manner of assessing the compensation and of enforcing claims, and also with the so-called "industrial" diseases, the inclusion of which forms so marked an advance on the older statutes.

Criticism of a principle which, for good or ill, is now recognised by all political parties as part and parcel of our social and economical system would be out of place; but the experience of former legislation on the same subject does not encourage hopes that the new Act will be any the less provocative of discussion and disputes as to the exact meaning and interpretation of its various sections; and the ideal of a compensation Act obnoxious to the ingenuity of neither lawyers nor insurance companies is still far to seek. The original draftsman of the Act may well plead that the innumerable amendments which are the result of prolonged debates render symmetry of form and expression impossible; but, even apart from this, the application of a wholly novel legal principle to infinitely varying circumstances within limits incapable of very exact definition must, with the best intentions in the world, necessarily produce a set of rules vague and often loosely expressed, the final interpretation of which, in spite of the most exhaustive artificial definitions, must probably lie with the Courts.

One fact, however, emerges with very great clearness, and that is the necessity which will be imposed on almost every kind of employer to insure against the risks created by the Act. The exact limits of these risks cannot of course at present be accurately defined, but they are sufficiently large to prevent anyone taking the chance of finding himself outside them. The effect of the Act may be summarised as follows :—

### Who are Liable to Pay Compensation?

All "employers" of the various kinds of workmen as defined hereafter are liable. The word is used in its ordinary sense, and includes corporations and unincorporated bodies, such as partnerships and the like; executors of deceased employers or other legal representatives; and also persons who have hired from someone else the services of any workman.

### What is a Workman?

Every workman as defined by the Act may claim compensation in appropriate circumstances; and it is the very wide meaning given to the term which constitutes the almost revolutionary character of the Act. The term includes :—

(*a*) Every person employed under a contract of service *by way of manual labour*, whatever his remuneration may be. The phrase "manual labour" has been the occasion of much litigation, and the test applied by the Courts appears to be (in the words of a learned author) "whether the manual labour is the chief duty of the servant, or only a duty incidental and subsidiary to other and more important mental ones." Thus, a shop assistant and a railway guard are not manual labourers, while a porter is. The last two cases in particular well illustrate the dividing line.

(*b*) Every person under a contract of service (express or implied) with an employer by way of clerical work *or otherwise*, whose remuneration does *not* exceed £250 a year. This seems so wide as to admit of practically no exceptions (save a few mentioned in the Act itself and referred to below), and to include almost everyone who works for a master. Domestic servants probably form as large a number as any other class who benefit by the section, but the importance of it also to clerks, shop assistants, and the like does not need to be pointed out; and it seems that even a curate may now claim compensation from his vicar. Hospital nurses are clearly within its scope, but a more difficult question may arise with regard to medical men, holiday appointments at hospitals, and other places whose salary does not exceed the prescribed limits; probably they are included, and it is difficult to see why the words of the Act should not be so read, although the result at first sight appears a little incongruous and perhaps ludicrous; and it must of course be remembered that, in their case, "accidents" in respect of which a claim could be made would not be common.

(*c*) Masters of ships and seamen who are otherwise "workmen" within the meaning of the Act. The addition of this class is a complete novelty. It does *not* include :—

(*a*) A person whose employment is of a casual nature and who is employed otherwise than for the purposes of the employers' trade or business. Perhaps a charwoman called in from time to time would be as good an example as any, or again, a person employed to clean the windows of a factory in which he does not usually work.

(*b*) Outworkers; who are defined in the Act as persons "to whom articles or materials are given out to be made up, cleaned, washed, altered, ornamented, finished or repaired, or adapted for sale in their own home or on other premises not under the control or management of the person who gave out the materials or articles."

(*c*) Members of the employer's family, dwelling in his house.

(*d*) Members of a police force.

(*e*) Persons in the military or naval service of the Crown.

### Who May Benefit by the Compensation?

Primarily, of course, the workman himself; but if the accident has proved fatal, other considerations apply. In such a case the workman's "dependents" have a right to be compensated. "Dependents" here means "members of his family as were wholly or in part dependent upon the earnings of the workman at the time of his death." Illegitimate children and grandchildren and the parents and grandparents of an illegitimate workman are (for the first time) included. "Members of the family" means wife, husband, father, mother, grandparents, and step-parents, children, grandchildren, step-children, brother and sister, half-brother and sister.

Dependency is a question of fact, often giving rise to very difficult problems. A son who puts his weekly wages into a common household fund has been held to have persons in part dependent on him; and the law has, in this direction (such being, in the view of the Court of Appeal, the intention of the Legislature), been usually construed as far as possible in favour of the workman.

### In Respect of what Claims May be Made.

Compensation is payable in the event of "personal injury by accident arising out of and in the course of" the workman's employment. These words have given rise to an enormous number of cases under the former Acts. What is an "accident"? And where is the line to be drawn with respect to the words "arising out of and in the course of"? The former is said (by the House of Lords) necessarily to include the idea of something sudden, fortuitous, and unexpected, and it was for this reason that diseases contracted owing to the nature of the employment have hitherto been held not to be accidents, because it was impossible to indicate the actual moment at which they began. This is remedied in the new Act, which directs that compensation may be claimed for injury to health caused by anthrax, lead, mercury, phosphorus and arsenic poisoning, and ankylostomiasis, the Home Secretary having power to add to these from time to time. The diseases must be contracted in the course of certain scheduled processes, and certified as so contracted as directed by the Act, and the employer liable is he who last employed the workman during the twelve months previous to his disablement (which is regarded as the date of the "accident") in the employment which has caused the disease, unless such employer can prove as a fact that it was caused while the workman was employed elsewhere in the same period. Contributory negligence on the workman's part affords no defence to the employer; and even that which the Act calls "serious and wilful misconduct" is only a bar where death or serious and permanent disablement results. This has slightly extended the law, which may now press very hardly on employers. A mere breaking of the employer's rules is not serious and wilful misconduct, but a deliberate refusal to employ necessary and prescribed safeguards, as well as drunkenness, might well be so.

### How is the Claim to Compensation Enforced?

First, the workman must give notice to his employer of the accident as soon as practicable and *before* the workman has voluntarily left the employment in which he was injured and (except in case of mistake, absence from England, or other reasonable cause) a delay in giving it of six months is fatal to the claim.

The whole question is then referred to an arbitrator, who is to be the County Court judge, if the parties cannot agree on one. Appeals in points of law lie from the arbitrator to the County Court judge and thence to the Court of Appeal. A schedule to the Act contains minute directions as to these arbitrations, and it is particularly to be noticed that a judge may now sit with a medical assessor.

The employer may require the workman who has given notice of an accident to be examined by a medical man chosen by the employer, and a refusal by the workman to submit to such examination suspends his claim to compensation for the time being. The employer or the workman may also demand further examinations from time to time, when the compensation has taken the form of a weekly payment, to ascertain if the health of the workman and his capacity for work have improved or otherwise. If the report of the medical man is considered unsatisfactory by either, the matter may be referred on the application of both parties to a medical referee, whose decision shall be final. It seems possible that the necessity of a joint application for such a reference may give rise to difficulties.

How is the Compensation to be Assessed?

It is to be noticed in the first place that no compensation is in any event payable unless the injury disables the workman from earning full wages at his employment for at least one week; and secondly, that if the workman is so disabled for less than two weeks, no compensation is payable in respect of the first week. This proviso is intended to prevent malingering or false claims. Before the new Act no compensation was payable for the first fortnight, but now after a fortnight it will be seen that the payment relates back to the accident itself. Clearly there will be a temptation to extend the period of incapacity over fourteen days, and the proviso in the Act admittedly gives only a rough working rule, and in fact represents a compromise arrived at during the parliamentary debates. But there seems no reason why it should not work as well as many others.

The maximum compensation in any case is £300, and is computed as follows:—When death results, dependents *wholly* dependent on the workman's earnings receive a sum equal to his average earnings for three years *or* £150, whichever is the larger, that is, up to £300. If the dependents are *partially* dependent, there is a proportionate deduction. If there are no dependents at all, the employer pays medical and funeral expenses with a limit of £10. Where total or partial incapacity results, the workman receives a weekly sum during the incapacity not exceeding 90 per cent. of his average weekly earnings during the preceding twelve months: unless he is under twenty-one, in which case, if his earnings are less than a pound a week, he receives not exceeding 100 per cent. of the same.

Allowance in all cases is to be made for payments or benefits which the workman may receive from the employer; and in the case of the payment of the lump sum to dependents, weekly payments previously made (before death) are to be deducted.

"Earnings" are by no means the same thing as "wages"; many other things may be taken into account, such as board and lodging, and the computation may often be one of great difficulty, the decision in all cases resting with the arbitrator.

The amount of the weekly payment may be varied by the County Court judge from time to time according to circumstances on the application of either party.

Finally, after running for six months, the weekly payment may be commuted by the employer for lump sum, calculated in the manner provided by the Act. Neither this sum nor any weekly payment may be assigned, charged, or attached.

Such are the main features of the Act which (it should be observed) preserves intact all rights given to the workman by the common law or the Employers' Liability Act, 1880. These chiefly deal with questions of personal negligence on the part of the employer or his managers and the like, and are important in that no limit whatsoever is fixed to the sum which a jury may award to a successful plaintiff.

It is not to be supposed that the present is the last Compensation Act which we shall see; but its provisions should be found comprehensive enough to satisfy for some years at all events the great army of "workmen"; and perhaps it may be prophesied that the next step will rather be in the direction of compulsory insurance by all employers, a necessary complement (it would seem) to the new legislation.

